# Combinatorial Analysis of CD4^+^Tregs, CD8^+^Teffs, and Inflammatory Indices Predict Response to ICI in ES-SCLC Patients

**DOI:** 10.3390/cancers18020192

**Published:** 2026-01-07

**Authors:** Anastasia Xagara, Konstantinos Tsapakidis, Vassileios Papadopoulos, Alexandros Kokkalis, Evangelia Chantzara, Chryssovalantis Aidarinis, Alexandros Lazarou, George Christodoulopoulos, Matina Perifanou-Sotiri, Dimitris Verveniotis, Vasiliki Rammou, Maria Smaragdi Vlachou, Galatea Kallergi, Alexandra Markou, Ioannis Samaras, Filippos Koinis, Emmanouil Saloustros, Athanasios Kotsakis

**Affiliations:** 1 Laboratory of Oncology, Faculty of Medicine, School of Health Sciences, University of Thessaly, Mezourlo, 41221 Larissa, Thessaly, Greece; xagaraa@hotmail.com (A.X.); tsapakidisk@uth.gr (K.T.); vasilispapadopoulos1@hotmail.com (V.P.); alexkokkalis@hotmail.gr (A.K.); valiaxantzara@gmail.com (E.C.); valadisaidarinis@yahoo.com (C.A.); lazaroualexander@gmail.com (A.L.); gchristodoulopoulos@hotmail.gr (G.C.); matina95d@gmail.com (M.P.-S.); vervedim@gmail.com (D.V.); vas19ram@gmail.com (V.R.); smarovlachou96@gmail.com (M.S.V.); alemarkou@uth.gr (A.M.); jnsamaras@gmail.com (I.S.); fkoinis@uth.gr (F.K.); esaloustros@uth.gr (E.S.); 2 Department of Medical Oncology, University General Hospital of Larissa, Mezourlo, 41221 Larissa, Thessaly, Greece; 3 Laboratory of Biochemistry/Metastatic Signaling, Section of Genetics, Cell Biology and Development, Department of Biology, University of Patras, 26504 Rion, Achaia, Greece; gkallergi@upatras.gr

**Keywords:** small cell lung cancer, immunotherapy, predictive biomarkers, regulatory T cells, Treg/Teff ratio, neutrophil-to-lymphocyte ratio, eosinophils, blood signature

## Abstract

**Background**: Small-cell lung cancer (SCLC) is an aggressive type of lung cancer, and several factors are currently used to predict poor outcomes, including performance status (PS), extensive-stage disease, male sex, advanced age, and elevated lactate dehydrogenase (LDH) levels. In this study, we aimed to explore the role of Tegs and inflammatory indices, such as CRP and NLR, in predicting response to immunotherapy. **Methods**: Fifty-one therapy-naïve ES-SCLC patients and ten healthy donors were enrolled. Peripheral blood mononuclear cells (PBMCs) were isolated and stained with fluorochrome-conjugated monoclonal antibodies. Multicolor flow cytometry was performed to determine the levels of CD8^+^ T cells and CD4^+^ Tregs, as well as their correlation with inflammatory indices and clinical outcomes. **Results:** ES-SCLC patients harbored higher percentages of CD8^+^ Teffs (*p* = 0.005) and FOXP3^+^ Tregs (*p* < 0.0001) in circulation before therapy compared with healthy donors. In addition, high levels of CD3^+^CD8^+^ T effectors were associated with longer PFS (*p* = 0.018) and longer OS (*p* = 0.012) compared with patients bearing low levels, while Tregs were not found to be predictive. More importantly, a survival benefit was observed in ES-SCLC patients with a low Treg/Teff ratio, as longer OS was observed in those with high percentages of CD8^+^ Teffs and low FOXP3^+^CTLA-4^+^ Tregs (*p* = 0.014) compared with those bearing low CD8^+^ Teffs and high FOXP3^+^CTLA-4^+^ Tregs. A low Treg/Teff ratio was further associated with low eosinophil levels and a low NLR before treatment initiation. **Conclusions**: These findings suggest a novel, easily obtainable blood-based signature that may help predict response to ICIs in ES-SCLC patients.

## Simple Summary

Small-cell lung cancer is a very aggressive form of lung cancer, and only a portion of patients benefit from new treatments that activate the immune system. Doctors currently lack simple tests that can show in advance which patients are more likely to respond. In this study, we examined routine blood measurements and specific immune cells circulating in the blood. We found that patients with a lower balance of certain regulatory immune cells compared with active immune cells, along with lower levels of inflammation markers, tended to live longer after starting immunotherapy. Our results suggest that a simple blood-based profile may help identify which patients are more likely to benefit from treatment, offering a practical tool for guiding therapy decisions and improving patient care.

## 1. Introduction

Lung cancer is ranked as the second-most frequently diagnosed cancer and is the leading cause of cancer-related deaths. Small-cell lung cancer (SCLC), which is typically more aggressive, accounts for 14% of all lung cancer cases [1]. Patients diagnosed with limited-stage small-cell lung cancer (SCLC) have a life expectancy of less than 24 months, while the majority of patients with extensive-stage SCLC, despite the available treatments, typically survive for no longer than 12 months [1,2]. There are several factors that can be used to predict a poor outcome, including performance status (PS), extensive-stage disease, male sex, advanced age, and high levels of lactate dehydrogenase (LDH) [2,3,4].

In certain forms of cancer, there are pre-existing inflammatory conditions before the development of malignancy [5]. On the other hand, in other types of cancer, the presence of oncogenic changes triggers an inflammatory environment that supports tumor growth [6]. Irrespective of its cause, persistent inflammation in the tumor microenvironment has various effects that promote tumor growth [6]. Further evidence suggests that heightened systemic inflammation correlates with a less favorable prognosis across multiple solid tumor types [7]. Several markers, like C-reactive protein (CRP), Glasgow Prognostic Score, neutrophil-to-lymphocyte ratio (NLR), and platelet-to-lymphocyte ratio (PLR), are linked with poor prognosis [8,9,10,11]. These markers have also been associated with immunotherapy, which has been approved as a highly effective treatment option for various types of cancers [12].

There are numerous predictive biomarkers used to predict which tumors are more likely to respond to ICIs, like programmed death ligand 1 (PD-L1) expression and tumor mutational burden (TMB) [13]. In this context, various types of cells play a crucial role in regulating the immune response. One such cell type is regulatory T cells (Tregs), which possess the ability to suppress immune responses and exert influence on immunotherapy [14]. Finally, there is a medical need for predictive biomarkers that can be easily obtained, like CRP and neutrophil-to-lymphocyte ratio (NLR), as the absolute counts of neutrophils and lymphocytes could potentially serve as indicators of the balance between pro-tumoral inflammation and anti-tumoral immune responses [15,16].

In this study, we explored the role of Tregs in predicting immunotherapy in untreated ES-SCLC. Moreover, we evaluated the potential of inflammatory hematological indices, such as NLR and CRP levels, to predict responders.

## 2. Materials and Methods

### 2.1. Patients and Blood Collection

Fifty-one therapy-naïve patients diagnosed with extensive SCLC and fifteen matched healthy donors (HDs) were enrolled in this study. The eligibility criteria were (i) age > 18 years, (ii) histologically confirmed diagnosis of SCLC, (iii) extensive clinical stage, and (iv) no prior line of therapy. The median age of the patients was 70 years old (44–84 years). All of them were treated with first-line ICI therapy combined with carboplatin and etoposide according to NCCN guidelines. Of these patients, 35.3% had liver metastases, while 29.4% had lung metastases (Table 1). Response Evaluation Criteria in Solid Tumors (RECISTs) were used to assess response to therapy.

Peripheral blood was collected in K2 ethylenediaminetetraacetic acid (EDTA; BD Biosciences, Heidelberg, Germany) from all patients at baseline, before any treatment. Levels of CRP and NLR were recorded before treatment and before the fourth cycle of therapy. The study was conducted in line with the Ethical Principles for Medical Research Involving Human Subjects, according to the World Medical Association Declaration of Helsinki. All patients provided written informed consent in order to participate in the study, which was approved by the local ethics and scientific committees of the University General Hospital of Larissa, 41334 Larissa, Greece (32710/3-8-20, approval on 25 August 2020).

### 2.2. Lymphocyte Isolation and Flow Cytometry Analysis

Peripheral blood mononuclear cells (PBMCs) were isolated from all patients and healthy donors with Hypaque-1077 (Sigma-Aldich, St. Louis, MO, USA). Isolated PBMCs were then re-suspended in RPMI-1640 medium (Biosera, Nuaille, France), that was supplemented with heat-inactivated fetal bovine serum (10%) (Gibco, Grand Island, NY, USA) and penicillin and streptomycin (1%) (Solarbio, Beijing, China). Freezing of PBMCs was performed in a freezing mix containing RPMI-1640 supplemented with 20% FBS and 10% DMSO (Sigma-Aldrich, UK), and the cells were stored at −80 °C until flow cytometric analysis.

Staining of PBMCs for the detection of surface markers was performed as previously described [17]. Briefly, the following anti-human fluorochrome-conjugated monoclonal antibodies were used: anti-CD3 PE-Cy7; anti-CD4 BV510; anti-CD8 APC-Cy7; anti-CD45RA PE; anti-CD45RO APC; anti-CCR7 FITC for T lymphocytes; and anti-CD3 PE-Cy7; anti-CD4 BV510; anti-FoxP3 FITC; anti-CD25 PerCpCy5.5; anti-CD127 BV421; anti-CTLA-4 APC for Tregs (all antibodies were purchased from Biolegend, San Diego, CA, USA). Staining buffer consisted of PBS supplemented with 1% BSA, and staining was performed for 30 min on ice in the dark. BD FACSChorus v3.0 Software on a Melody flow cytometer (BD Biosciences, Heidelberg, Germany) was used for acquisition of the different T-cell populations. For each measurement, 10^6^ single-cell events were counted. Negative controls consisted of unstained cells, and FMO-stained cells were used for setting the gates (Supplementary Figure S1).

### 2.3. Statistical Analysis

For statistical analysis, GraphPad Prism version 10 (GraphPad Institute Inc., San Diego, CA, USA) was used. To corelate immune cell phenotypes with patients’ clinical outcomes, Kaplan–Meier analysis was performed together with the log-rank test. For this type of analysis, percentages of different T-cell types were divided into high and low groups according to receiver operating characteristic (ROC) curves cut-offs (Supplementary Figure S2). Overall survival (OS) was calculated as the number of days from patient enrollment until death or last follow-up, while progression-free survival (PFS) was defined as the number of days from patient enrolment to disease relapse or death, whichever occurred first. The nonparametric Mann–Whitney U test was used to determine differences between groups. Moreover, Pearson’s chi-square test and Fisher’s exact test were used to analyze changes in inflammatory signatures between patient groups. Differences and associations were considered significant when *p* < 0.05. All *p*-values were two-sided.

## 3. Results

### 3.1. CD8^+^ T Effectors and CD4^+^ Tregs in the Peripheral Blood of SCLC Patients

**Figure 1:**
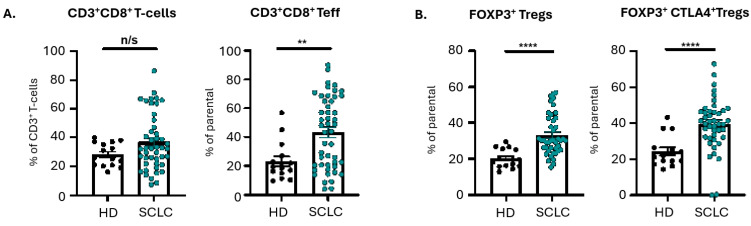
Percentages of different subpopulations of CD8^+^ T cells and CD4^+^ Tregs in the circulation of therapy-naïve SCLC patients and healthy individuals. (**A**) Graphs show the frequencies of CD3^+^CD8^+^ T cells and CD3^+^CD8^+^CD45RA^+^CD45RO^−^CCR7^−^ (T effectors), and (**B**) of CD3^+^CD4^+^CD25^+^CD127^−^FOXP3^+^ (FOXP3^+^Tregs) and CD3^+^CD4^+^CD25^+^CD127^−^FOXP3^+^CTLA-4^+^ (FOXP3^+^CTLA-4^+^Tregs). HD: healthy donors (*n* = 15); SCLC: extensive-stage SCLC (*n* = 51); n/s: non-significant; **: *p* < 0.01; ****: *p* < 0.0001.

PBMCs from fifty-one treatment-naïve SCLC patients having ES-SCLC and fifteen healthy donors were analyzed by multicolor flow cytometry. SCLC patients had significantly higher percentages of CD8^+^ T effectors (*p* = 0.005), but not CD8^+^ T cells (*p* = 0.189), compared with healthy individuals. Additionally, SCLC patients had higher percentages of FOXP3^+^ Tregs (*p* < 0.0001) and FOXP3^+^ Tregs expressing CTLA-4 (*p* < 0.0001) compared with healthy donors (Figure 1).

### 3.2. Correlation of CD8^+^ T Effectors and CD4^+^ Tregs with Clinical Outcome

**Figure 2:**
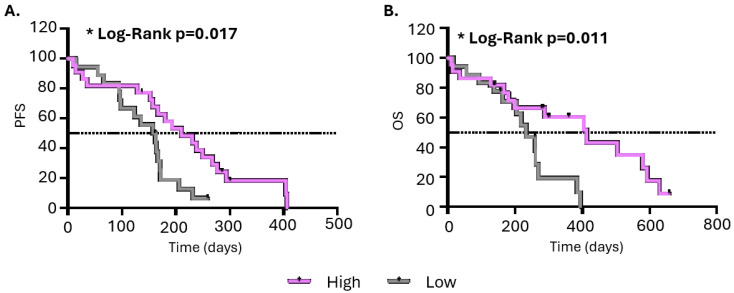
Kaplan–Mayer analysis for CD8^+^Teffs of (**A**) PFS and (**B**) OS. Purple line: patients with high percentages of CD8^+^ Teffs in the circulation; gray line: patients with low percentages of CD8^+^ Teffs in the circulation. PFS: progression-free survival; OS: overall survival; * *p *< 0.005.

To investigate if any of the T-cell populations were correlated with clinical outcome, patients were separated into two groups bearing high or low cell populations using the cut-offs defined by ROC curves. Results indicated that high levels of CD3^+^CD8^+^ T effectors in the circulation of SCLC patients before therapy were associated with longer PFS (median: 210 vs. 161 days; *p* = 0.018) and longer OS (median: 412 vs. 232 days; *p* = 0.012) compared with patients bearing low levels. Other subtypes of CD8^+^ T cells and CD4^+^ Tregs were not found to be correlated with PFS or OS (Table 2 and Figure 2).

Additionally, correlation analysis was performed between CD8^+^ or CD8^+^ T effectors and different subtypes of CD4^+^ Tregs. CD8^+^ T cells were negatively correlated with FOXP3^+^ Tregs (Spearman r: −0.363, *p* = 0.018) but not with CTLA-4^+^ Tregs (Spearman r: −0.067, *p* = 0.684). Moreover, correlation analysis between CD8^+^T effectors and CD4^+^ Tregs indicated a significant negative correlation between CD8^+^T effectors and CTLA-4^+^ Tregs (Spearman r: −0.365, *p* = 0.021), but not with FOXP3^+^ Tregs (Spearman r: −0.086, *p* = 0.597) (Table 3).

**Figure 3:**
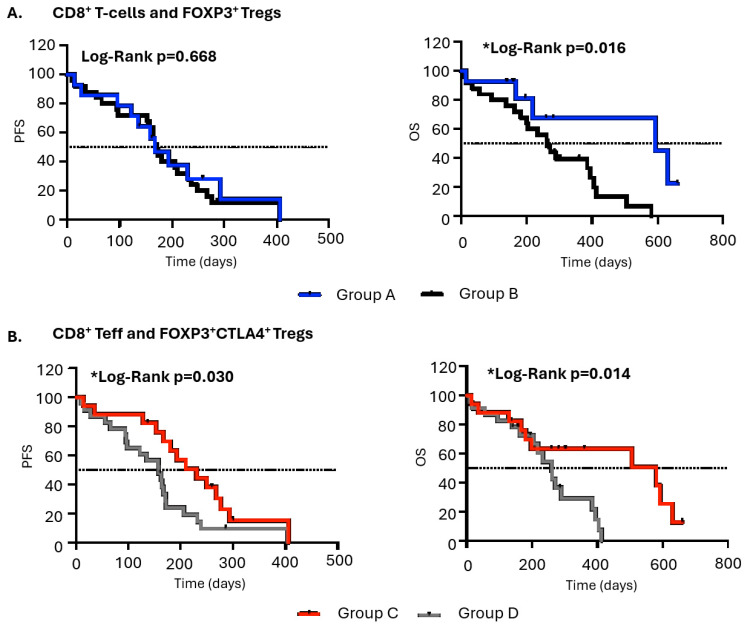
Prognostic significance of CD3^+^CD8^+^ T cells and Tregs in patients with extensive-stage SCLC. Kaplan–Meier plots of PFS and OS in patients separated into groups as follows: (**A**) group A, high CD8^+^ T cells (ROC cut-off) with low FOXP3 Tregs (*n* = 18); group B, the rest of the patients (*n* = 33); and (**B**) group C, high CD8^+^ T effectors (ROC cut-off) with low FOXP3^+^CTLA-4^+^Tregs (*n* = 21); group D, the rest of the patients (*n* = 30); * *p* < 0.05.

As CD8^+^ T cells were found to be negatively correlated with FOXP3^+^ Tregs and CD8^+^T effectors with CTLA-4^+^ Tregs, patients were separated into groups as follows: for CD8^+^ T cells, group A consisted of patients with high CD8^+^ T cells (ROC cut-off) and low FOXP3^+^ Tregs (ROC cut-off) (*n* = 18), and group B included the rest of the patients (*n* = 33); for CD8^+^T effectors, group C consisted of patients with high CD8^+^ T effectors (ROC cut-off) and low CTLA-4^+^ Tregs (ROC cut-off) (*n* = 21), and group D included the rest of the patients (*n* = 30). Regarding CD8^+^ T cells, patients in group A were not correlated with PFS (*p* = 0.668; med: 167 days vs. 171 days; HR 0.85) but had significant longer OS (*p* = 0.016; med: 592 days vs. 261 days; HR 0.35) compared with group B. Additionally, patients harboring high levels of CD8^+^T effectors and low levels of CTLA-4^+^ Tregs (group C) indicated significantly longer OS (*p* = 0.014; med: 580 days vs. 260 days; HR 0.42) and longer PFS (*p* = 0.030; med: 230 days vs. 158 days; HR 0.49) compared with patients in group D (Figure 3)**.**

### 3.3. Correlation of Inflammatory Signatures with Immunotherapy Response in SCLC Patients

**Figure 4:**
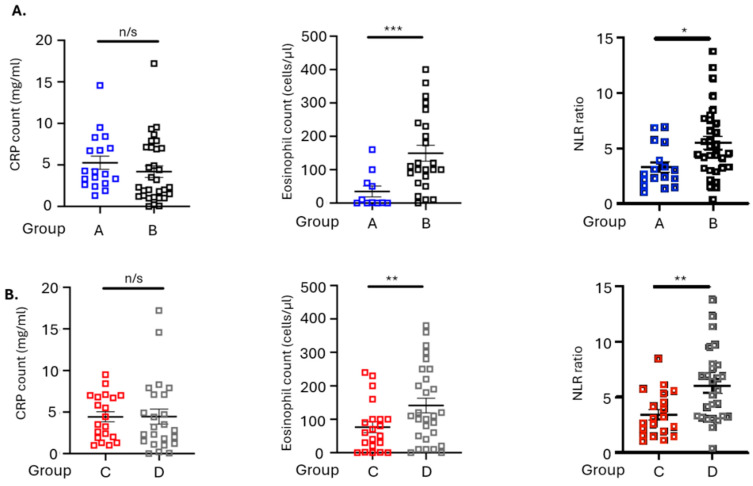
Levels of inflammatory signatures before treatment in SCLC patients separated into those having high numbers of CD8^+^ T cells and low numbers of FOXP3^+^ Tregs (group A) and the rest of them (group B), as well as those bearing high percentages of CD8^+^T effectors and low numbers of CTLA-4^+^ Tregs (group C) and the rest of them (group D). Graphs depicting the levels of NLR, CRP, and eosinophil counts (**A**) between groups A and B and (**B**) between groups C and D. n/s: non-significant; * *p* < 0.05, ** *p* < 0.005; *** *p* < 0.0001.

As high percentages of CD8^+^T cells and low percentages of Tregs (group A), and high percentages of CD8^+^ Teffs with low percentages of CTLA4^+^ Tregs (group C), in circulation found to be predictive for survival under first-line ICI therapy, we next proceeded to analyze patients’ inflammatory status. Levels of inflammatory markers, including neutrophil-to-lymphocyte ratio (NLR), CRP, and eosinophils, were counted before treatment initiation and after the third therapy cycle. Kaplan–Mayer analysis of PFS and OS in patients bearing high or low baseline levels of inflammatory markers did not reveal any significant differences (Supplementary Figure S3). At baseline, patients in in group A had significantly lower levels of NLR (*p* = 0.0101) and eosinophil counts (*p* = 0.0006) compared with patients in group B, whereas CRP levels (*p* = 0.1406) did not differ between the two groups (Figure 4A). Moreover, patients in group C had significantly lower levels of NLR (*p* = 0.0398) and eosinophil counts (*p* = 0.0006), while CRP levels did not differ (*p* = 0.6033), compared with patients in group D (Figure 4B).

**Figure 5:**
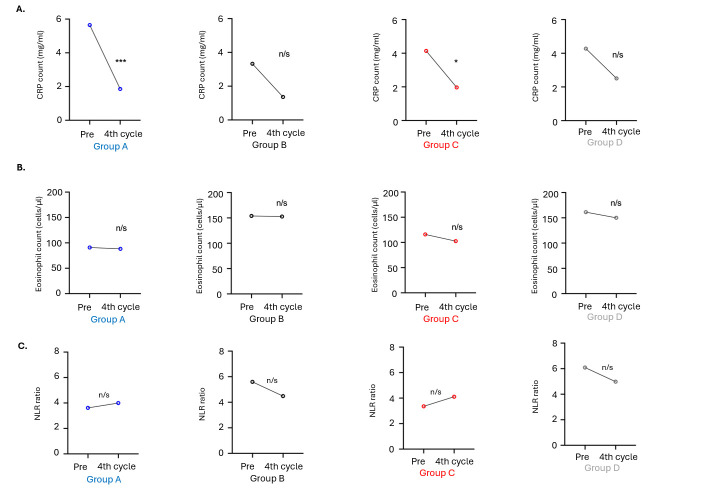
Graphs depicting median changes of (**A**) CRP, (**B**) eosinophils, and (**C**) NLR during immunotherapy treatment. Patients were separated into those having high numbers of CD8^+^ T cells and low numbers of FOXP3^+^ Tregs (group A) and the rest of them (group B), as well as those bearing high percentages of CD8^+^T effectors and low numbers of CTLA-4^+^ Tregs (group C) and the rest of them (group D). n/s: non-significant; * *p* < 0.05; *** *p* < 0.0001.

Changes in inflammatory markers from baseline (pretreatment) to end of the third cycle were also examined in a total of 29 patients (11 patients in group A/C and 18 patients in group B/D) for whom data were available at both time points. The results indicated a significant reduction in CRP levels from baseline to the end of the third cycle in patients in group A (*p* = 0.0005) and group C (*p* = 0.0244), but not in patients in group B (*p* = 0.086) and group D (*p* = 0.188). Levels of eosinophils and NLR were not found to have significant changes during immunotherapy in any of the four groups tested (Figure 5, Supplementary Figure S4).

## 4. Discussion

SCLC patients comprise a population with significant morbidity and mortality. For many years, treatment options were limited. Recently, immunotherapy has been approved for extensive-stage disease in the first-line setting. However, important questions remain regarding which category of patients benefits the most, highlighting the need for predictive biomarkers. Additionally, the role of assessing peripheral T-cell phenotypes and inflammatory indices in predicting overall survival (OS) after ICI treatment in SCLC is unclear.

In the present study, we explored the predictive value of inflammatory cells and indices in the circulation of extensive-stage SCLC patients receiving first-line immunotherapy. The results indicated higher levels of circulated T effectors and Tregs in extensive-stage SCLC patients than in healthy controls, as has been described previously [18]. A significant negative correlation between T effectors and FOXP3^+^CTLA-4^+^ Tregs, and between CD8^+^ T cells and FOXP3^+^ Tregs, in circulation before treatment initiation was observed and was further correlated with OS. Furthermore, in patients with longer OS during immunotherapy, we detected significantly lower levels of NLR and eosinophil counts before treatment initiation. CRP levels were significantly decreased during immunotherapy treatment only in the group of patients with longer OS.

Tregs are subsets of CD4^+^ T cells that express the transcription factor FoxP3 and favor tumorigenesis [19,20]. Their immunosuppressive activity is mediated by various mechanisms, thereby affecting a wide range of different immune cell types [21]. Tregs can both reduce the number and function of Teffs by secreting granzyme B, perforin, and cytokines such as IL-10 [22]. Additionally, they express high levels of inhibitory receptors such as PD-1 and CTLA-4, thereby affecting antigen presentation and reducing APC function [23]. In this study, we detected elevated levels of FOXP-3 Tregs and CTLA-4^+^FOXP-3^+^ expressing Tregs in the circulation of treatment-naïve ES-SCLC patients. Elevated levels of Tregs in circulation have been detected in various tumor types and have been correlated with worse clinical outcomes [24,25]. In SCLC, Treg levels were not predictive of ICI response. FOXP-3^+^ Tregs were also not predictive for ES-SCLC patients receiving chemotherapy, as described previously by others [18]. To our knowledge, this has not been previously described for ES-SCLC patients receiving first-line immunotherapy.

The role of CD8 T cells in targeting and eliminating cancer cells is indispensable. Teffs comprise a category of differentiated antigen-specific T cells that express perforin and are cytotoxic [26,27]. They also express ICI inhibitors such as PD-1 and CTLA-4 in response to persistent stimulus by tumor cells, which reduces their cytotoxic activity, a state called T-cell exhaustion [28]. In this study, we observed high levels of CD8^+^ Teffs in the circulation of SCLC patients compared with healthy individuals. These high levels were predictive of response to ICI regarding PFS and OS. However, further studies are needed to validate the predictive value of circulating Teffs in ICI response in SCLC patients.

Significantly, we observed a strong negative correlation between CD8^+^ Teffs and CTLA-4^+^ Tregs in circulation, which was reflected in longer OS for the subgroup of patients harboring high levels of CD8^+^ Teffs and low levels of CTLA-4^+^ Tregs. This was also the case for patients harboring high percentages of CD8^+^ T cells and low levels of FOXP3^+^ Tregs. The Treg/Teff ratio has previously been shown indicate the balance between immunosuppression and immunoactivation in cancer [29]. It has also been mentioned as a marker for disease staging in SCLC, as patients with limited-stage disease (LS-SCLC) seem to harbor a lower Treg/Teff ratio compared with ES-SCLC patients [30]. Mechanistically, it remains unclear how the balance between these two immune cell populations is regulated; however, multiple biological events play pivotal roles in their interaction [31]. Tregs direct regulate Teff cells by suppressing their function in the TME through the secretion of inhibitory cytokines (e.g., TGF-β) or by reducing their population through cytotoxic molecules (e.g., perforin) [32]. Teff metabolism is also regulated by antagonistic consumption of IL-2 by Tregs [33]. In addition, direct cell–cell interactions, mediated mainly by checkpoint inhibitors expressed on Tregs (e.g., CTLA-4), reduce the activation and activity of Teff cells [33]. Currently, the rationale for the prognostic use of this ratio is weak, as it indicates a favorable outcome in some cancer types and an unfavorable one in others [34]. To our knowledge, this study is the first to describe that a low Treg/Teff ratio is a favorable predictive factor for ES-SCLC receiving first-line ICI therapy.

Chronic inflammation promotes tumor growth and, subsequently, patients’ inflammatory state measured by blood inflammatory markers comprises a promising, easily obtained predictive tool [35]. Such measurable indices with predictive value in different cancer types include levels of the NLR, CRP, and eosinophil count [12]. None of the indices examined in this study were predictive for response to ICI at baseline. In addition, levels of the NLR and eosinophil count were significantly lower in patients in groups A and C, who were shown to have longer OS. Moreover, CRP levels were significantly reduced in both groups during ICI treatment, indicating that early changes in CRP during immunotherapy treatment could be used as a surrogate marker for early monitoring of response. This has recently been shown to be the case for other tumor types, such as NSCLC [36].

## 5. Conclusions

Ιn this study, we explored the predictive value of various inflammatory signatures for immunotherapy treatment in ES-SCLC patients. The data indicated higher percentages of both Teff and Treg cell populations before treatment in patients compared with healthy individuals. Importantly, a survival benefit in patients harboring a low Treg/Teff ratio, which was further accompanied by low eosinophil levels and a low NLR, a result that probably reflects an immunostimulatory environment. Although significant results are presented in the current exploratory study, several limitations exist, mainly related to the small number of patients. Future studies in larger multicenter cohorts are required to confirm, validate, and strengthen the statistical power of our findings. Moreover, due to the nature of this study, we were not able to exclude the possibility that the reduction in CRP levels during treatment is related to confounders such as infections or tumor burden. Overall, further validation in a larger cohort of ES-SCLC patients, as well as LS-SCLC patients, may offer an easily obtainable tool to select immunotherapy responders in daily clinical practice.

## Figures and Tables

**Table 1:** SCLC patients’ clinical characteristics.

Characteristic	Patients (%)
Age (median)	70 (44–84)
Sex	
Men	42 (82.3%)
Women	9 (17.7%)
Race	
Caucasian	51 (100%)
Metastatic sites	
Lung	15 (29.4%)
Brain	13 (25.5%)
Liver	18 (35.3%)
Bones	9 (17.6%)
Adrenal gland	6 (11.7%)
LNs	8 (15.7%)
Pleural	16 (31.4%)
other	1 (1.9%)
Best response	
PR	31 (60.7%)
SD	7 (13.7%)
Mixed	2 (3.9%)
N/A	11 (21.7%)

**Table 2:** Associations of different T-cell phenotypes and clinical outcome of treatment-naïve SCLC patients.

			PFS			OS		
T-Cell Populations	ROC Cut-Off		Median (Days)	95% HR CI	*p*-Value	Median (Days)	95% HR CI	*p*-Value
CD3^+^CD8^+^	31	High	172	0.475 to 1.852	0.851	385	0.259 to 1.282	0.144
		Low	169			246		
CD8^+^ Teffs	44	High	210	0.231 to 0.998	**0.018**	412	0.182 to 0.977	**0.012**
		Low	161			232		
FOXP3^+^ Tregs	29	High	171	0.500 to 1.819	0.884	266	0.672 to 2.896	0.362
		Low	167			261		
FOXP3^+^CTLA4^+^ Tregs	39	High	164	0.644 to 2.367	0.512	261	0.772 to 3.514	0.149
		Low	167			385		

Bold formatting indicates statistical significance (*p* < 0.05).

**Table 3:** Correlations of CD8^+^ T-cell populations with different CD4^+^ Treg populations.

		CD4^+ ^Tregs	
T Cells		FOXP3+	FOXP3^+^CTLA4^+^
CD8^+^	Spearman r	−0.363	−0.067
	*p *-value	**0.0181**	0.684
	95% CI	−0.606 to −0.057	−0.383 to 0.262
CD8^+^ Teffs	Spearman r	−0.086	−0.365
	*p *-value	0.597	**0.021**
	95% CI	−0.395 to 0.240	−0.613 to −0.0503

## Data Availability

The data presented in this study are available on reasonable request from the corresponding author.

## Supplementary Materials

The following supporting information can be downloaded at: https://www.mdpi.com/article/10.3390/cancers18020192/s1, Supplementary Figure S1: Gating strategy for Tregs detection. Supplementary Figure S2: ROC curves and AUG values of Teffs and FOXP3^+^Tregs. Supplementary Figure S3: Kaplan–Mayer curves for PFS and OS in patients bearing high percentages of (A) CRP levels, (B) eosinophil levels, and (C) NLR. Patients were separated into high and low groups using the median values. Supplementary Figure S4: Graphs depicting the changes of (A) CRP, (B) eosinophils, and (C) NLR during immunotherapy treatment for all patients. Patients separated into those having high numbers of CD8^+^ T cells and low numbers of FOXP3^+^ Tregs (group A) and the rest of them (group B), as well as those bearing high percentages of CD8^+^T effectors and low numbers of CTLA-4^+^ Tregs (group C) and the rest of them (group D).

## Abbreviations

The following abbreviations are used in this manuscript:

APC	Antigen-presenting cell
BD	Becton Dickinson
CD	Cluster of differentiation
CRP	C-reactive protein
CTLA-4	Cytotoxic T-lymphocyte-associated protein 4
DMSO	Dimethyl sulfoxide
EDTA	Ethylenediaminetetraacetic acid
ES-SCLC	Extensive-stage small-cell lung cancer
FACS	Fluorescence-activated cell sorting
FBS	Fetal bovine serum
FITC	Fluorescein isothiocyanate
FMO	Fluorescence minus one
FoxP3	Forkhead box P3
ICI	Immune checkpoint inhibitor
LDH	Lactate dehydrogenase
NLR	Neutrophil-to-lymphocyte ratio
NSCLC	Non-small-cell lung cancer
OS	Overall survival
PBMCs	Peripheral blood mononuclear cells
PBS	Phosphate-buffered saline
PFS	Progression-free survival
PLR	Platelet-to-lymphocyte ratio
PS	Performance status
RECIST	Response evaluation criteria in solid tumors
ROC	Receiver operating characteristic
SCLC	Small-cell lung cancer
TMB	Tumor mutational burden
Tregs	Regulatory T cells
