# Supplementary material for: Combinatorial Analysis of CD4+Tregs, CD8+Teffs, and Inflammatory Indices Predict Response to ICI in ES-SCLC Patients
Source: Cancers (Basel). 2026 Jan 7;18(2):192. doi: 10.3390/cancers18020192 (PMC12838880; doi:10.3390/cancers18020192)
Supplement: Supplementary file 1 [file cancers-18-00192-s001.zip › cancers-4058379-supplementary.pdf]

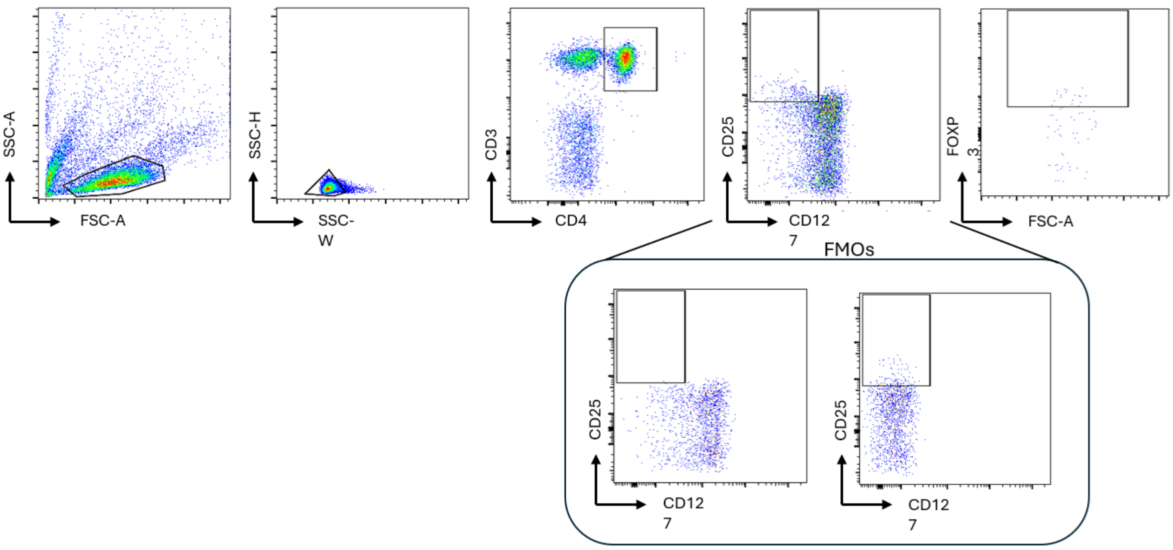

Supplementary Figure S1: Gating strategy for Tregs detection.

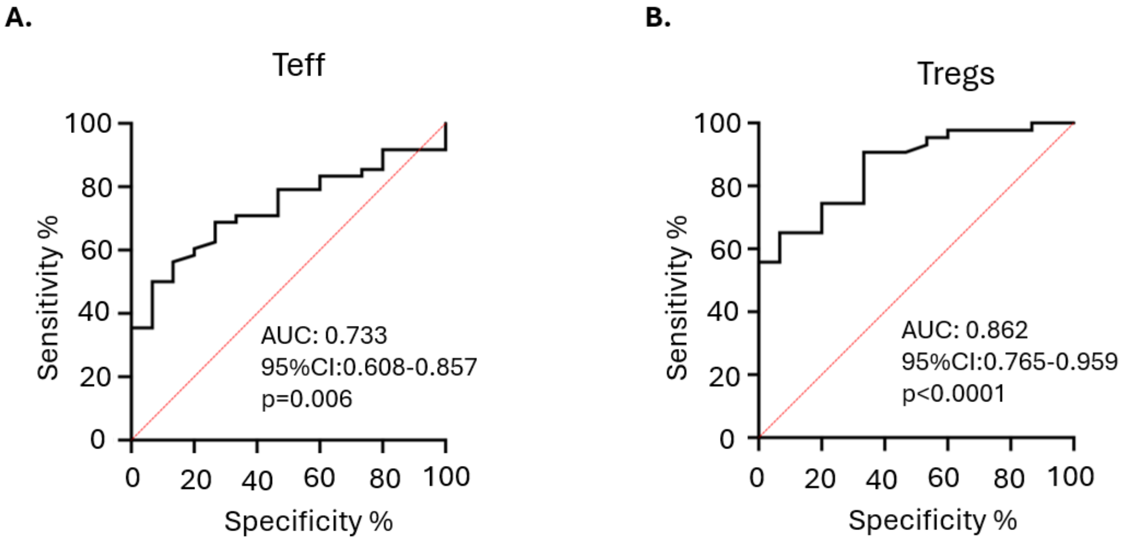

Supplementary Figure S2: ROC curves and AUG values of Teffs and FOXP3^{+}Tregs.

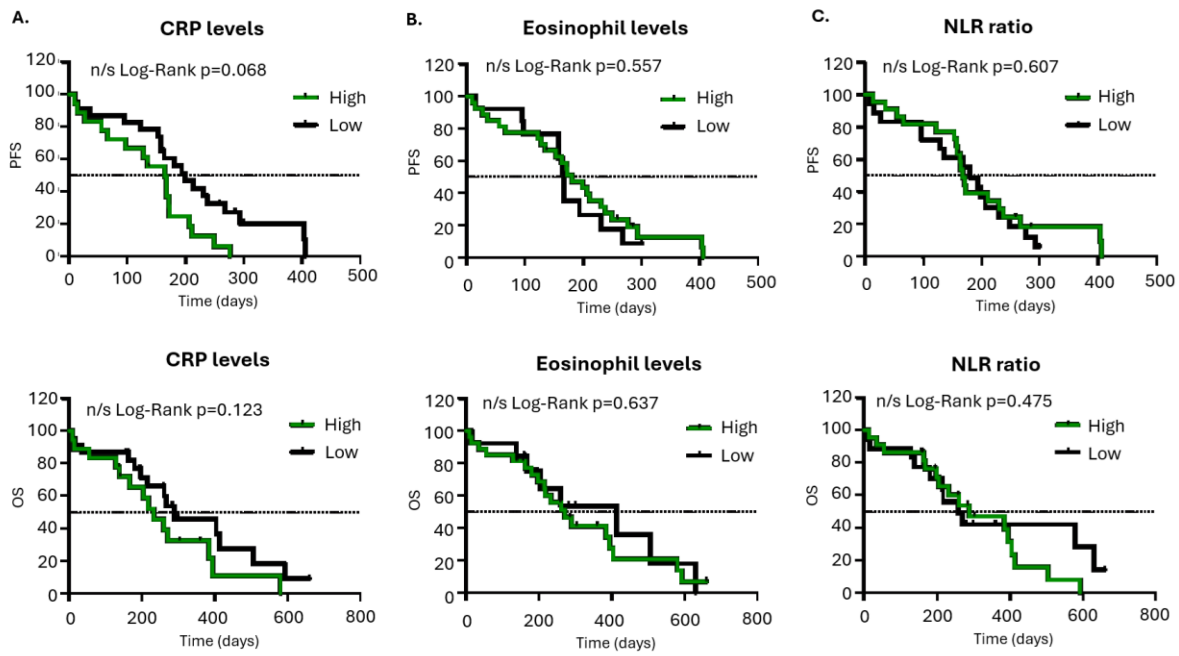

Supplementary Figure S3: Kaplan–Mayer curves for PFS and OS in patients bearing high percentages of (A) CRP levels, (B) eosinophil levels, and (C) NLR. Patients were separated into high and low groups using the median values.

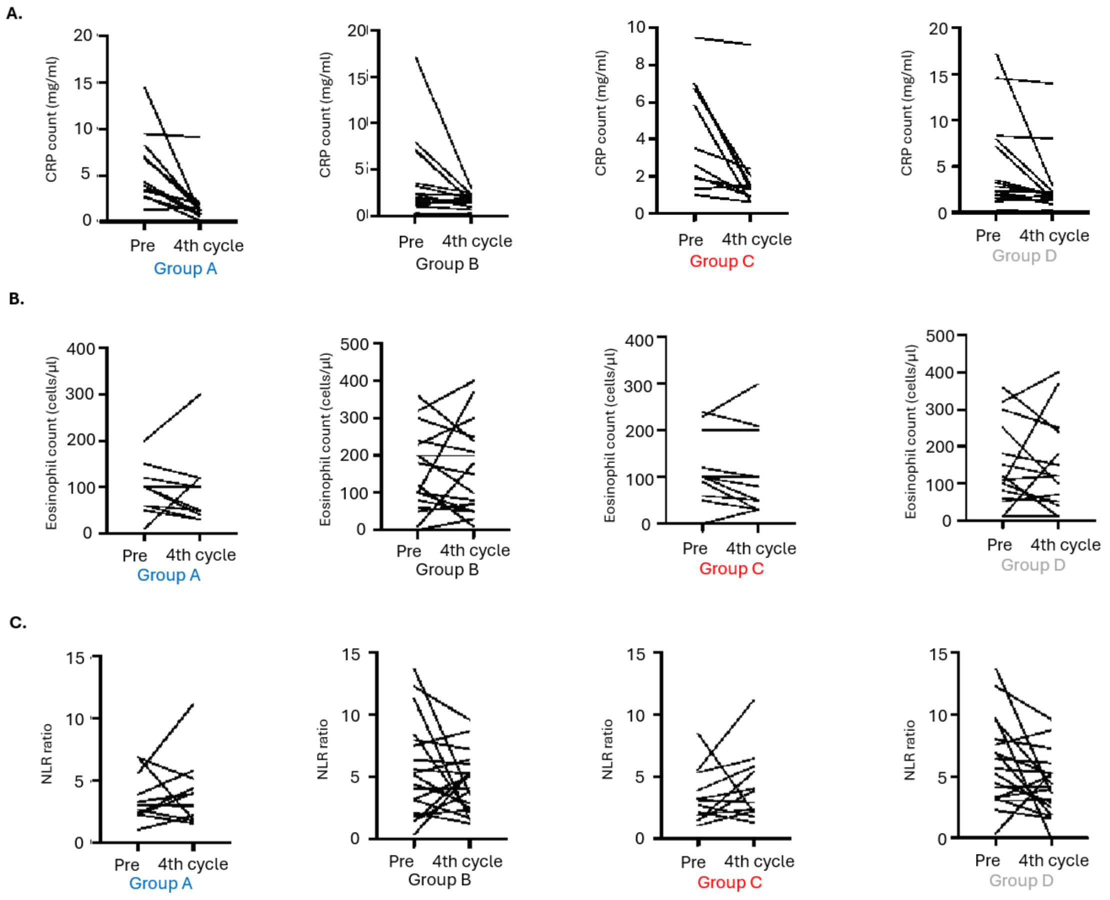

Supplementary Figure S4: Graphs depicting the changes of (A) CRP, (B) eosinophils, and (C) NLR during immunotherapy treatment for all patients. Patients separated into those having high numbers of $CD8^+$ T cells and low numbers of $FOXP3^+$ Tregs (group A) and the rest of them (group B), as well as those bearing high percentages of $CD8^+$T effectors and low numbers of CTLA-4^+ Tregs (group C) and the rest of them (group D).
